# Microarray expression profile of circular RNAs in lung tissues from rats with lipopolysaccharide-induced acute respiratory distress syndrome

**DOI:** 10.1016/j.dib.2017.10.018

**Published:** 2017-10-10

**Authors:** Qiquan Wan, Di Wu, Qifa Ye

**Affiliations:** a Department of Transplant Surgery, The Third Xiangya Hospital, Central South University, No 138 Tongzipo Road, Changsha, China; b Zhongnan Hospital of Wuhan University, Institute of Hepatobiliary Diseases of Wuhan University, Transplant Center of Wuhan University, Hubei Key Laboratory of Medical Technology on Transplantation, Wuhan, Hubei 430071, China

**Keywords:** Microarray, Circular RNAs, Lipopolysaccharide, Acute respiratory distress syndrome

## Abstract

The data presented in this article are related to the research article entitled “The expression profiles of circRNAs in lung tissues from rats with lipopolysaccharide-induced acute respiratory distress syndrome (ARDS): A microarray study.” (Wan et al., 2017) [1]. ARDS remains a common and devastating syndrome. The development of circRNA microarray has facilitated the study of the role of circRNAs in regulating gene expression in ARDS. This research was designed to explore the expression profile of circRNAs in lung tissues from rats with lipopolysaccharide-induced ARDS. The field dataset is made publicly available to enable critical or extended analyzes.

**Specialization Table**

**Table:** 

Subject area	Chemistry/Biology
More specific subject area	Rat with ARDS
Type of data	Table
How data was acquired	Arraystar Rat CircRNA Array (8×15K, Arraystar)
Data format	Raw and analyzed
Experimental factors	Lung tissues from 3 rats with lipopolysaccharide-induced ARDS vs. 3 normal rat lung tissues
Experimental features	Microarray expression profile analysis of circRNAs in rats with lipopolysaccharide-induced ARDS
Sample source location	Changsha, China
Data accessibility	Data are available via a web application (https://www.ncbi.nlm.nih.gov/geo/info/linking.html)

**Value of the data**

• We first performed microarray detection on the expression profiles of circRNAs in lung tissues from rats with lipopolysaccharide-induced ARDS. The data are important and can be consulted by the other researchers in future research in this research area.
• Using circRNA microarray data, the other researchers can compare the circRNA expression profiles in ARDS lung tissues with others methods.
• This data allows the other researchers and research students to extend the statistical analyses.

## 1 Data

The dataset of this article provides information on the expression profiles of circRNAs in lung tissues from rats with lipopolysaccharide-induced ARDS. Microarray and sample annotation data were deposited in Gene Expression Omnibus under accession number GSE102523. Direct link to deposited data is https://www.ncbi.nlm.nih.gov/geo/query/acc.cgi?acc=GSE102523.

## 2 Experimental design, materials and methods

### 2.1 Tissue samples and RNA preparation

Lung tissue samples were collected from Sprague Dawley rats undergoing lipopolysaccharide-induced ARDS and normal rats [1, 2]. Total RNA was isolated from 3 ARDS rat lung tissue samples and 3 normal rat lung tissues using TRIzo Reagent (Invitrogen, USA).

### 2.2 Labeling and hybridization

Sample labelling and microarray hybridization were performed according to the Arraystar's standard protocols (Arraystar, Rockville, Maryland, USA) [1]. In short, total RNA was treated with Ribonuclease R (RNase R) (Epicenter, Madison, WI, USA) to remove linear RNAs. Then, enriched circRNAs were amplified and transcribed into fluorescent cRNA using a random priming method based on Arraystar Super RNA Labeling protocol. After the concentration and specific activity were measured using a NanoDrop ND-1000, the labelled cRNAs (pmol Cy3/μg cRNA) were then hybridized onto the Arraystar Rat circRNA Arrays (8×15K, Arraystar). The circRNA expression microarray slides were incubated for 17 hours at 65 °C in an Agilent Hybridization Oven. After washing the slides, the hybridized arrays were fixed and scanned with an Agilent G2505C Scanner.

It is worthy of note that part of the probes in rat chip were designed by using mouse and human orthology sequence, hoping to find similar circRNAs in rat through the junction site on account of High Gene homology.

### 2.3 Microarray and quality control

The acquired scanned images were imported into Agilent Feature Extraction software (version 11.0.1.1) for raw data extraction. The R software package limma package (R version 3.1.2) was used for quantile normalization and subsequent data processing. After quantile normalization of the raw data, low intensity filtering was performed, and the circRNAs that at least 2 out of 6 samples have flags in “P” or “M” (“All Targets Value”) were retained for further analyses. CircRNAs exhibiting *P*-value less than 0.05 and fold change greater than 2.0 were considered as significantly differentially expressed circRNAs.

**Fig. 1:**
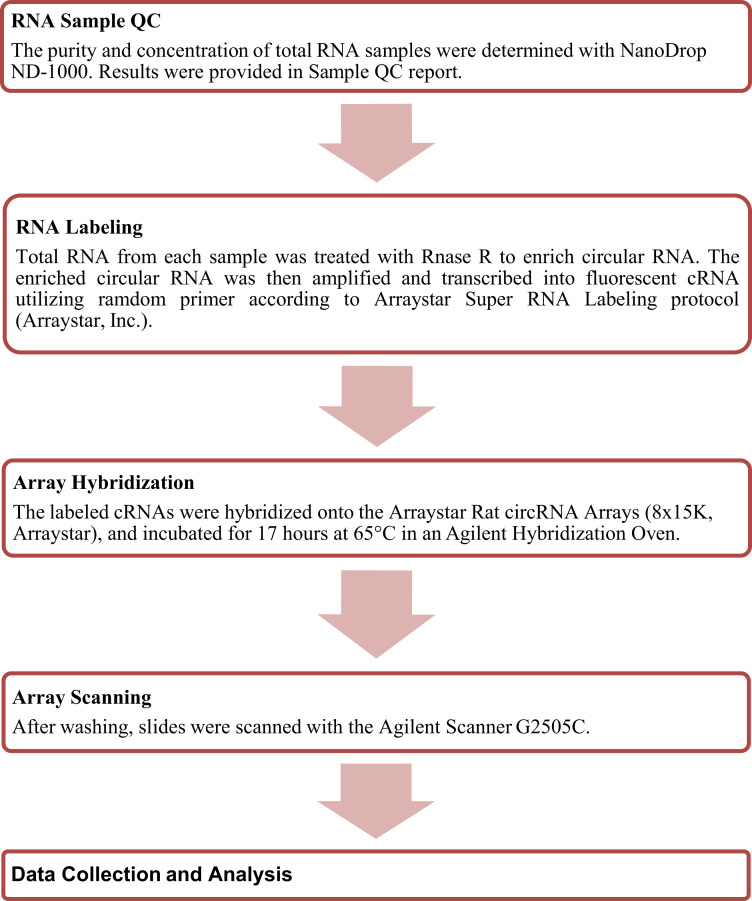
Experiment workflow of microarray expression profile of circular RNAs.

The experiment workflow is listed in Fig. 1.
