# Supplementary material for: Microarray expression profile of circular RNAs in lung tissues from rats with lipopolysaccharide-induced acute respiratory distress syndrome
Source: Data Brief. 2017 Oct 10;15:591–4. doi: 10.1016/j.dib.2017.10.018 (PMC5655385; doi:10.1016/j.dib.2017.10.018)
Supplement: Supplementary file 1 — Supplementary material [file mmc1.docx]

The authors declare that there are no conflicts of interest.
